# Common adult psychiatric disorders in Swedish primary care where most mental health patients are treated

**DOI:** 10.1186/s12888-017-1381-4

**Published:** 2017-06-30

**Authors:** Jan Sundquist, Henrik Ohlsson, Kristina Sundquist, Kenneth S. Kendler

**Affiliations:** 1 Center for Primary Health Care Research, Lund University, Malmö, Sweden; 2 Department of Psychiatry, Virginia Commonwealth University, Richmond, VA USA; 3 Department of Human and Molecular Genetics, Virginia Commonwealth University, Richmond, VA USA; 4 Virginia Institute for Psychiatric and Behavioral Genetics, Virginia Commonwealth University, Richmond, VA USA

**Keywords:** Mental disorders, Mental health, Personality, Population characteristics

## Abstract

**Background:**

The overall aim of this study is to present descriptive data regarding the treated prevalence of nine common psychiatric and substance use disorders in the first Primary Care Registry (PCR) in Sweden: Major Depression (MD), Anxiety Disorders (AD), Obsessive-Compulsive Disorder (OCD), Adjustment Disorder (AdjD), Eating Disorders (ED), Personality Disorder (PD), Attention Deficit Hyperactivity Disorder (ADHD), Alcohol Use Disorder (AUD) and Drug Abuse (DA).

**Method:**

We selected 5,397,675 individuals aged ≥18. We examined patterns of comorbidity among these disorders and explored the association between diagnoses in the PCR and diagnoses obtained from Hospital and Specialist care. We explored the proportion of patients with these nine disorders that are only treated in primary health care.

**Results:**

For four of our disorders, 80% or more of the cases were present *only* in the PCR: AdjD, DA, AD and MD. For two disorders (OCD and ED), 65–70% of cases were only found in the PCR. For three disorders (PD, AUD, and ADHD), 45–55% of the patients were only present in the PCR.

**Conclusion:**

The PCR will, in the future, likely prove to be an important tool for studies in psychiatric epidemiology.

## Background

Mood disorders, anxiety disorders, and stress- and adjustment disorders together account for a large portion of the disease burden in Western countries. In Sweden, according to a 2009 [1] study, depression and anxiety were among the 25 diseases with the highest insurance costs. In the 2010 Global Burden of Disease Study, depression and anxiety were ranked second and seventh in their worldwide impact on disease-related disability [2]. Most of these patients are likely seen by a general practitioner (GP), although a previous Swedish study [3] suggested that only 25% of the cases of depression and anxiety are identified in primary health care in the country.

There are limited nationwide studies in western countries on incidence and prevalence of common psychiatric disorders in primary health care. However, it is probable that a large proportion of such disorders are treated only in primary health care. Two questionnaire-based studies [3, 4], where data were collected in waiting rooms in primary health care centres in Sweden, observed that between 3.7 and 6.2% of patients had symptoms of depression; between 11.8 and 13.5% had symptoms of anxiety. Studies [5, 6] conducted in the United Kingdom, which has a highly developed primary health care system, estimated the incidence of depression to be 13.9 and anxiety to be 9.7 per 1000 person-years. In Belgium [7], incidence rates of depression diagnosed in primary health care were found to be 7.2 in men and 14.4 in women, per 1000 person-years. In a previous Swedish study [8], the 12-month prevalence of clinically diagnosed mood, anxiety and stress- and adjustment disorders was 2.4% (3.2% in women and 1.5% in men). The authors used data from a regional primary health care register from Stockholm, Värmland, Uppsala and Gotland comprising of 75 primary health care centres. None of these studies estimated the proportion of cases that were only treated in primary care.

Swedish registries have provided a tremendous resource for the study of the epidemiology and genetic epidemiology of psychiatric disorders [9–14]. However, up until now, the ascertainment of individuals with psychiatric disorders has been restricted to hospital-based diagnoses using the Hospital Discharge Register and specialist out-patient diagnoses from the Outpatient Care Register.

### Aims of the study

The first aim of this study is to present descriptive data regarding the treated prevalence of nine relatively common psychiatric and substance use disorders in the first Primary Care Registry (PCR) available in Sweden, which now covers approximately 72% of the Swedish population. The PCR is a research dataset including nationwide longitudinal individual based information on diagnoses and time for diagnoses; providing valuable information on the occurrence and course for common psychiatric disorders and could be linked to several different Swedish population-based registers that have nationwide coverage.

The nine common psychiatric and substance use disorders were: Major Depression (MD), Anxiety Disorders (AD), Obsessive-Compulsive Disorder (OCD), Adjustment Disorder (AdjD), Eating Disorders (ED), Personality Disorder (PD), Attention Deficit Hyperactivity Disorder (ADHD), Alcohol Use Disorder (AUD) and Drug Abuse (DA). We chose these disorders because they would often be seen and cared for in primary care settings as opposed to the rarer and more severe disorders such as bipolar illness, schizophrenia or autism that would usually be treated in speciality settings. The second aim is to examine patterns of comorbidity among these disorders, and explore the association between diagnoses in the PCR and i) diagnoses obtained from the Hospital and Specialist care, ii) treatment with common classes of psychopharmacologic agents, iii) personal and parental educational status and iv) marital status. Third, we attempt to validate these disorders in several ways including resemblance in full siblings (as a long established method for validating psychiatric disorders [15]) treatment with specific classes of psychopharmacological agents, social class of origin, and educational and marital status. By validation, we mean evidence that having the diagnosis predicts additional meaningful clinical information external to the diagnostic process. Finally, we demonstrate the proportion of patients with nine common psychiatric disorders that are only treated in primary health care.

## Methods

The Primary Care Register (PCR) is a research dataset including individual-level information such as diagnoses based on visits to primary health care centres in the following regions in Sweden: Värmland (during 2005–2015), Uppsala Län (2005–2015), Västernorrland (2008–2015), Norrbotten (2001–2014), Halland (2007–2014), Skåne (1998–2013), Östergötland (1998–2014), Stockholm (2003–2016), and Västra Götaland (2000–2013). The time periods are different depending on the time before digitalising the patient records. In 2015 these nine counties contained 72% of the population living in Sweden. In total there were 208,624,743 registrations from 7,372,164 individuals. The aim is to include almost all of the Swedish population into the PCR. Each registration included the unique individual Swedish 10-digit personal ID number, which is assigned at birth or immigration to all Swedish residents, date of the visit, and the ICD 10 code. The ID number was replaced by a random number to preserve confidentiality. The PCR could be linked to several different Swedish population-based registers with national coverage (i.e. Hospital Discharge Register, Outpatient Care Register, Prescribed Drug Register and the Multi-Generation Register). We secured ethical approval for this study from the Regional Ethical Review Board of Lund University (No. 2008/409).

From the PCR we selected all individuals with at least one registration at age 18 or older (*N* = 5,397,675). We identified all individuals with at least one of nine diagnoses defined in Table 1. Based on the tetrachoric correlation matrix (Table 2), we used an exploratory factor analysis (EFA) to identify the underlying relationships between the nine diagnoses. The purpose with the factor analysis is to describe the variability and patterns of comorbidity among the nine psychiatric and substance use disorders by a small number of factors.

**Table 1:** Individuals registered by diagnostic category at least once in the PHC-database 1998 and onwards and 18 years or older at first registration

Diagnosis	ICD-codes	Number registered (%) Total *N* = 5,397,675	Sex ratio in prevalence (females/males)
Major Depression	F32, F33	670,980 (12.4%)	1.90 (1.89; 1.91)
Anxiety Disorders	F40, F41	536,279 (9.9%)	1.81 (1.80; 1.82)
Obsessive-Compulsive Disorder	F42	17,941 (0.3%)	1.42 (1.38; 1.46)
Adjustment Disorder	F43	498,960 (9.2%)	2.37 (2.36; 2.39)
Eating Disorders	F50	12,633 (0.2%)	10.33 (9.70; 11.01)
Personality Disorder	F60, F61	16,743 (0.3%)	1.59 (1.54; 1.64)
Attention Deficit Hyperactivity Disorder	F90	25,891 (0.5%)	0.81 (0.79; 0.83)
Alcohol Use Disorder	F10	109,415 (2.0%)	0.49 (0.39; 0.40)
Drug Abuse	F11-F19	142,118 (2.6%)	0.98 (0.97; 0.99)

**Table 2:** Tetrachoric correlations (± SEs) and ORs (with 95% confidence intervals) between different diagnoses^a^

	MD	AD	OCD	AdjD	ED	PD	ADHD	AUD	DA
Major Depression (MD)	1	10.0 (9.9; 10.1)	8.3 (8.1; 8.6)	6.5 (6.5; 6.6)	8.3 (8.0; 8.6)	14.1 (13.6; 14.5)	7.8 (7.6; 8.0)	4.2 (4.1; 4.2)	3.4 (3.4; 3.5)
Anxiety Disorders (AD)	0.64 (0.01)	1	16.0 (15.5; 16.5)	5.8 (5.8; 5.9)	9.7 (9.3; 10.0)	19.0 (18.4; 19.6)	10.0 (9.7; 10.2)	4.7 (4.6; 4.7)	4.0 (4.0; 4.1)
bsessive-Compulsive Disorder (OCD)	0.41 (0.00)	0.52 (0.00)	1	4.1 (4.0; 4.2)	18.8 (17.4; 20.3)	26.0 (24.5; 27.6)	15.3 (14.4; 16.3)	3.6 (3.4; 3.8)	3.0 (2.8; 3.1)
Adjustment Disorder (AdjD)	0.53 (0.00)	0.49 (0.00)	0.26 (0.00)	1	4.6 (4.5; 4.8)	7.2 (7.0; 7.4)	4.8 (4.6; 4.9)	2.3 (2.3; 2.4)	2.6 (2.5; 2.6)
Eating Disorders (ED)	0.40 (0.00)	0.42 (0.00)	0.40 (0.01)	0.28 (0.00)	1	35.2 (33.1; 37.4)	13.9 (12.9; 14.9)	3.9 (3.7; 4.2)	2.9 (2.8; 3.1)
Personality Disorder (PD)	0.50 (0.00)	0.55 (0.00)	0.47 (0.01)	0.37 (0.00)	0.51 (0.01)	1	50.1 (48.0; 52.2)	12.7 (12.2; 13.2)	11.3 (10.9; 11.7)
Attention Deficit Hyperactivity Disorder (ADHD)	0.41 (0.00)	0.46 (0.00)	0.40 (0.01)	0.31 (0.00)	0.37 (0.01)	0.60 (0.00)	1	10.1 (9.7; 10.4)	11.0 (10.7; 11.3)
Alcohol Use Disorder (AUD)	0.34 (0.00)	0.36 (0.00)	0.19 (0.00)	0.19 (0.00)	0.20 (0.01)	0.42 (0.00)	0.40 (0.00)	1	10.4 (10.3; 10.6)
Drug Abuse (DA)	0.30 (0.00)	0.33 (0.00)	0.17 (0.00)	0.22 (0.00)	0.16 (0.01)	0.41 (0.00)	0.43 (0.00)	0.49 (0.00)	1

^a^Correlations below diagonal and ORs above

We then used the same ICD codes, as defined in Table 1, to identify individuals with a registration in the Specialist Outpatient Care Register (during 2001–2012) and the Hospital Discharge Register (during 1969–2012). Furthermore, from the Prescribed Drug Register, we defined individuals that had collected at least one prescription of Antidepressants (ATC-code N06A), Anxiolytics (ATC-code N05B) and Stimulants (ATC-code N06B). This register records information on sales of prescribed pharmaceutical agents dispensed by the Swedish Corporation of Pharmacies.

### Analysis

To provide evidence of the potential validity of these diagnoses, we first studied the correlation among full sibling pairs for each diagnosis. In the PCR we identified 2,190,806 unique full sibling pairs (excluding twin pairs). Second, we studied the association between educational attainment and the diagnoses for all individuals born prior to 1990 (*n* = 5,141,890). The education variable was based on information from the education register the number of years of education (1:<9 years; 2: 9 years; 3: 10–11 years; 4: 12 years; 5: 13–15 years; 6: 16 years or more; 7: PhD/ licentiate degree; groups 5,6 and 7 include education at university level). The education register (UREG) holds statistics of the Swedish population educational level. In order to be able to compare the variable over time, we standardised the variable (mean 0 and std. 1) by gender and year of birth. Third, to measure the association between social class and diagnosis, we used mean parental education (measured the same way as explained above) as a proxy for social class. We were able to identify parental educational status in 3,366,585 individuals. Finally, we identified individuals that, sometime during the period 1998–2014, were registered as married as well as individuals that were registered as divorced. We studied the association between marital status (yes/no) and divorce (yes/no) and the above defined diagnoses using logistic regression while controlling for year of birth and gender. All statistical analyses were performed using SAS 9.3. [16]

## Results

Table 1 depicts the prevalence in our treated sample of each of the nine disorders of interest, which varied from a low of 0.2% for EDs to a high of 12.4% for MD. This table also shows the sex ratio in prevalence. Males have higher prevalence for three disorders (ADHD, DA and AUD) and females for the remaining six: MD, AD, OCD, AdjD, ED and PDs.

The observed patterns of comorbidity are seen in Table 2 both as ORs (above the diagonal) and tetrachoric correlations (below the diagonal). Focusing on the ORs, they were all significantly greater than unity and were particularly high between PDs and all other diagnoses, among AUD, ADHD and DA, among MD, AD and OCD, and between ED and OCD. The raw proportion and total numbers of individuals with each of these comorbidities is depicted in Table 3.

**Table 3:** – Patterns of comorbidities showing the raw proportions and total number of individuals with specific comorbid patterns of diagnoses

	MD	AD	OCD	AdjD	ED	PD	ADHD	AUD	DA
Major Depression (MD)		4.72%	0.17%	3.73%	0.12%	0.19%	0.24%	0.71%	0.81%
Anxiety Disorders (AD)	272,860		0.21%	2.99%	0.12%	0.20%	0.24%	0.65%	0.75%
Obsessive-Compulsive Disorder (OCD)	10,021	11,879		0.09%	0.01%	0.02%	0.02%	0.02%	0.02%
Adjustment Disorder (AdjD)	215,624	172,609	5481		0.07%	0.12%	0.15%	0.37%	0.51%
Eating Disorders (ED)	6984	6692	730	4169		0.02%	0.01%	0.02%	0.02%
Personality Disorder (PD)	11,202	11,374	1266	7077	1176		0.05%	0.06%	0.07%
Attention Deficit Hyperactivity Disorder (ADHD)	13,937	13,984	1213	8689	787	2978		0.08%	0.10%
Alcohol Use Disorder (AUD)	41,287	37,633	1264	21,393	957	3422	4455		0.38%
Drug Abuse (DA)	46,704	43,497	1343	29,643	941	3864	5971	22,122	

*% above diagonal and number below diagonal

We applied an exploratory factor analysis to a tetrachoric correlation matrix for all of the diagnoses. The scree plot had only one factor with an Eigen value of greater than one. Table 4 shows the loadings of that single factor, which were lowest for DA and AUD and highest for PD, AD and MD.

**Table 4:** Factor loadings on a single factor from an exploratory factor analysis

Disorder	Factor loadings
Major Depression	0.72
Anxiety Disorders	0.78
Obsessive-Compulsive Disorder	0.58
Adjustment Disorder	0.53
Eating Disorders	0.55
Personality Disorder	0.79
Attention Deficit Hyperactivity Disorder	0.68
Alcohol Use Disorder	0.50
Drug Abuse	0.49

We next examined the association between the diagnoses recorded in the PCR with those obtained from specialist and in-patient care (Table 5). These ORs were uniformly high but quite variable across the different disorders, and consistently higher between the PCR and specialist care than between the PCR and in-patient diagnoses. These ORs were much higher for four of the diagnostic categories (OCD, ED, PD and ADHD), which suggests that primary care GPs were especially likely to refer such cases to specialist or in-patient care. By contrast, these ORs were relatively low for MD, AD and AdjD.

**Table 5:** Sources of registration of cases in the primary care registry and the proportion treated with antidepressants, anxiolytics and stimulants

	OR (95% CI) for disorder in		Percent of PCR Cases that are			Percent of PCR cases that are prescribed		
Disorder	PCR and specialist care	PCR and inpatient	Only in PCR	Also in specialist care	Also in inpatient	Antidepressants	Anxiolytics	Stimulants
Major Depression	15.9 (15.8; 16.2)	10.3 (10.2; 10.5)	81.1%	16.9%	5.9%	79.4%	54.0%	2.6%
Anxiety Disorders	18.8 (18.6; 19.0)	11.7 (11.4; 11.9)	81.7%	17.2%	3.2%	67.4%	63.5%	2.9%
Obsessive-Compulsive Disorder	403.5 (387.2; 420.4)	190.1 (173.2; 208.7)	69.3%	30.0%	4.1%	79.2%	59.0%	6.0%
Adjustment Disorder	10.5 (10.4; 10.7)	5.6 (5.5, 5.7)	89.9%	8.8%	2.5%	48.6%	43.4%	1.9%
Eating Disorders	425.9 (407.2; 445.4)	257.8 (240.0; 277.1)	65.5%	32.6%	9.7%	66.7%	51.6%	6.3%
Personality Disorder	327.7 (316.7; 339.1)	117.3 (112.0; 123.0)	48.3%	49.0%	15.6%	80.1%	71.7%	14.6%
Attention Deficit Hyperactivity Disorder	344.3 (334.1; 354.6)	144.0 (133.3; 155.4)	53.6%	46.0%	4.3%	66.8%	56.9%	58.6%
Alcohol Use Disorder	136.2 (134.0; 138.5)	65.2 (64.2; 66.3)	46.1%	44.4%	33.6%	51.5%	50.9%	4.2%
Drug Abuse	49.4 (48.4; 50.5)	27.5 (26.9; 28.0)	82.3%	15.0%	11.2%	51.0%	44.3%	5.1%

We calculated the proportion of individuals with our nine disorders in the PCR who were also registered with the same diagnosis in the specialist and in-patient registries (Table 5). For four of our disorders, 80% or more of the cases were present *only* in the PCR: AdjD, DA, AD and MD. For two of the disorders (OCD and ED), 65–70% of cases were only found in the PCR. For the remaining three disorders (PD, AUD, and ADHD), 45–55% of the patients were only present in the PCR.

For AUD and DA, we were also able to compare our PCR diagnoses with registrations in the criminal registry. We found ORs of 25.22 (24.57–25.88) for AUD and 11.99 (11.79; 12.19) for DA.

We were also able to match our PCR cases with the pharmacy registry and show in Table 5 the proportion that had filled prescriptions for anti-depressants, anxiolytics and stimulants. Antidepressants prescriptions were uniformly high across all the diagnoses with the highest treatment rates seen in PD, MD and OCD. High rates of anxiolytics prescriptions were also widespread across these psychiatric disorders, with highest rates for PDs and ADs. Stimulants, by contrast, were rarely prescribed for all categories except ADHD and, more modestly, for PDs. We also show the percentage of patients prescribed these three classes of medication who were only registered in the PCR database (Table 6), thereby depicting the prescription patterns of the primary care physicians. They are relatively similar to those seen in all patients, although the rates are somewhat lower, especially for stimulant treatment of patients with ADHD.

**Table 6:** The proportion of cases only registered in the primary care registry that were treated with antidepressants, anxiolytics and stimulants

	Percent of PCR cases *only registered in PCR* that are prescribed		
Disorder	Antidepressants	Anxiolytics	Stimulants
Major Depression	75.9%	49.5%	1.7%
Anxiety Disorders	63.2%	59.6%	1.9%
Obsessive-Compulsive Disorder	73.6%	55.2%	5.1%
Adjustment Disorder	45.4%	40.4%	1.5%
Eating Disorders	60.8%	48.1%	5.6%
Personality Disorder	74.3%	65.0%	11.7%
Attention Deficit Hyperactivity Disorder	59.3%	49.7%	32.1%
Alcohol Use Disorder	46.1%	42.6%	2.8%
Drug Abuse	46.5%	38.6%	2.2%

Table 7 examines a range of additional potential validators for these common psychiatric diagnoses. All of the disorders were familial with tetrachoric correlations in full siblings ranging from +0.15 for MD and AdjD to +0.36 for ADHD. All of the diagnoses were associated with reduced educational attainment, with the association being strongest for ADHD and DA and weakest with AdjD. We indexed social class of origin by parental educational attainment and found that diagnoses of OCD, PD and ED were all associated with higher social class, while DA stood out as having by far the strongest association with a lower social class of origin.

**Table 7:** Siblings correlations, self- and parental-educational status and marital status by diagnosis

Disorder	Sibling correlations	Education – OR^a^	Mean parental education OR^b^	Married		Divorced	
				%	OR^c^	%	OR^c^
Major Depression	0.15 (0.00)	0.89 (0.89; 0.90)	0.97 (0.96; 0.97)	56.7	0.87 (0.86; 0.87)	25.3%	1.59 (1.58; 1.60)
Anxiety Disorders	0.18 (0.00)	0.83 (0.83; 0.84)	0.94 (0.94; 0.94)	53.5	0.77 (0.77; 0.78)	23.7%	1.46 (1.45; 1.47)
Obsessive-Compulsive Disorder	0.23 (0.01)	0.90 (0.89; 0.92)	1.06 (1.04; 1.08)	40.3	0.53 (0.52; 0.55)	16.9%	1.10 (1.06; 1.15)
Adjustment Disorder	0.15 (0.00)	0.95 (0.95; 0.96)	0.97 (0.97; 0.97)	61.3	1.16 (1.16; 1.17)	26.7%	1.88 (1.87; 1.90)
Eating Disorders	0.23 (0.01)	0.90 (0.89; 0.92)	1.11 (1.09; 1.13)	40.2	0.56 (0.54; 0.58)	18.6%	1.41 (1.34; 1.48)
Personality Disorder	0.29 (0.01)	0.69 (0.67; 0.70)	1.08 (1.06; 1.10)	33.6	0.39 (0.38; 0.41)	26.7%	2.02 (1.95; 2.09)
Attention Deficit Hyperactivity Disorder	0.36 (0.01)	0.58 (0.57; 0.59)	0.96 (0.95; 0.98)	36.2	0.50 (0.49; 0.51)	22.4%	1.88 (1.82; 1.95)
Alcohol Use Disorder	0.22 (0.00)	0.71 (0.71; 0.72)	0.92 (0.91; 0.93)	42.5	0.48 (0.47; 0.48)	34.1%	2.48 (2.45; 2.51)
Drug Abuse	0.22 (0.00)	0.60 (0.59; 0.60)	0.78 (0.77; 0.78)	49.1	0.64 (0.64; 0.65)	31.7%	2.16 (2.13, 2.18)
Population				60.0		18.4%	

^a^Education is standardized by Year of Birth and gender. Only individuals 25 years and older are included. The OR illustrates the decreased odds per 1 SD increase in education.

^b^Sample size *N* = 3,366,585 as parental educational status not available on the oldest members of the PCR. The OR illustrates the decreased odds per 1 SD increase in education.

^c^Controlling for year of birth and sex

Finally, we examined marital status and found that eight of our nine disorders (all but AdjD) were associated with a reduced probability of marriage (correcting for age) with the reduction being strongest for PD, ADHD and OCD. The probability of divorce was increased for all diagnostic categories, and was strongest for AUD, DA and PD and weakest for OCD.

## Discussion

This is the first paper to report that of all cases of AdjD, DA, AD and MD found in the primary health care register, 80% were not found in the outpatient/inpatient registers, indicating that a large majority of the psychiatric consultations occur in primary health care. A factor analysis of our results suggested that the pattern of comorbidity across these nine disorders could be explained by a single latent factor. We did not see evidence for separate factors of internalizing disorders (most typically anxiety and depression) and externalizing disorders (often ADHD and substance abuse syndromes) as has often been seen in other epidemiological interview based studies [17, 18].

We are also the first to demonstrate a novel association between psychiatric disorders in primary health care and specialist psychiatric care. Another interesting finding is the reasonable estimate for MD prevalence -- 12.4% -- well within the range reported previously from large population based survey studies [19] and only modestly lower than that reported from an interview based survey in neighbouring Norway [20]. We could also confirm that men have higher prevalence for ADHD, DA and AUD while women for ND, AD, OCD, AdjD, ED and PDs. This is in agreement with previous research [21] that women had elevated rates of anxiety and depressive disorders and that men had higher prevalence for drug and alcohol abuse.

We emphasise that PCR covers most of the visits to primary health care in Sweden. It is known that, across a two-year span, the entire Swedish population will have visited a primary health care centre regarding any health problem. Our finding that only a low proportion of the patients in PCR were also registered with the same diagnosis in the specialist and in-patient registries is also new. No such data has ever been presented previously. For four of our disorders, 80% or more of the cases were present only in the PCR: AdjD, DA, AD and MD, an expected finding that has now been confirmed.

The gender differences in this report (female: male ratio of 1.9) agreed with other studies demonstrating that female gender is one of the most consistent and stable factors associated with depression. An overall 1-year female/male ratio of 2/1 has been shown in the past [22, 23]. A population based epidemiologic study [19], using survey data from 10 different countries, also showed that rates of major depression were higher for women than men.

For two of the disorders (OCD and ED), 65–70% were only found in the PCR. This is an unexpected finding because such disorders might be expected to be treated predominantly in specialist psychiatric care. For the remaining three disorders (PD, AUD, and ADHD), 45–55% of the patients were only present in the PCR and would normally also be treated in specialist psychiatric care. Our findings imply that an increasing number of GPs may be taking care of their patients with psychiatric problems without seeking referral to specialist care.

We suggest introducing a new term, primary health care psychiatry, for the concept of treating such disorders in primary health care. Many of these centres already have psychologists and counsellors, as well as interested doctors, that would like to receive more specific training to aid in their treatment for these disorders.

It is not easy to compare our nationwide data on prevalence and incidence rates of ADHD, DA and AUD, MD, AD, OCD, AdjD, ED and PDs with those of previous studies [5–7]. For example, previous studies [3, 4] in primary health care settings in Sweden reported prevalence rates of MD of 12.5–19.7%. In a previous study [8], which included registry data from 75 primary health care centres in mid-Sweden (mostly Stockholm), the overall 12-month prevalence rate was 3.2% in women and 1.5% in men. However, previous studies [3, 4] used screening tools to identify symptoms of depression and anxiety in primary health care settings, from which they estimated the prevalence and incidence.

### Validation of the PCR

Firstly, our results support the validity of these diagnoses within the PCR and the potential value of this registry as a resource, through linkage to a wide array of other Swedish registries, for the study of the epidemiology and genetic epidemiology of common psychiatric disorders.

Secondly, our results support previous research that psychiatric diagnoses examined in this study are associated with low educational status [21, 24, 25] . In the PCR, all of the diagnoses were associated with reduced educational attainment, with the association being strongest for ADHD and DA and weakest with AdjD.

Third, we measured the association between social class operationalised as mean parental education and diagnose of interest as a proxy for social class and found that diagnoses of OCD, PD and ED were all associated with higher social class, while DA stood out as having by far the strongest association with a lower social class of origin. These findings were in agreement with other studies.

Fourth, in broad agreement with other studies [21, 24, 25] we found that nearly all of our nine disorders were associated with a decreased probability of marriage and an increased probability of divorce with the association with divorce being strongest for the drug use disorders of DA and AUD.

Fifth, we found for DA and AUD striking ORs for receipt of a diagnosis in the PCR and registration for the same condition in the criminal registry.

Finally, in the validation we found that all of the disorders were familial with tetrachoric correlations in full siblings ranging from +0.15 for MD and AdjD to +0.36 for ADHD, which was in broad agreement with prior comparable studies [26–30].

### Limitations and strengths

We analyse treated psychiatric disorders and are likely underestimating true population prevalence both because some individuals with psychiatric illness do not visit primary care and some who do will not report their psychiatric symptoms. We cannot rule out the presence of false-positive psychiatric diagnoses as primary care physicians will not typically have time to do comprehensive psychiatric evaluations of their patients. A major strength of this report is the large study population: 7,372,164 persons. To the best of our knowledge no other study of this size has previously been performed in primary health care. Data on the study population were obtained from Swedish counties and regions and their primary health care databases, which contain clinical information about all adult individuals registered at 75 different primary health care centres. Thus, the findings are likely to reflect the current situation in primary health care in Sweden.

## Conclusions

This paper shows novel findings based on a new national primary health care research database, PCR, demonstrating that a large majority of all out-patient physician contacts for AdjD, DA, AD and MD occur in primary health care, and a reasonable estimate for MD prevalence; 12.4%, and confirm that women men ratio in MD is 1.9. An unexpected finding, OCD and ED, 65–70%, were only found in the PCR. The PCR will be an important tool for future studies in psychiatric and substance abuse epidemiology and genetics.

## Abbreviations

AD: Anxiety disorders
ADHD: Attention deficit hyperactivity disorder
AdjD: Adjustment disorder
AUD: Alcohol Use disorder
DA: Drug abuse
ED: Eating disorders
MD: Major depression
OCD: Obsessive-compulsive disorder
PCR: Primary care registry
PD: Personality disorder
